# Maternal resources for care are associated with child growth and early childhood development in Bangladesh and Vietnam

**DOI:** 10.1111/cch.12911

**Published:** 2021-09-22

**Authors:** Sulochana Basnet, Edward A. Frongillo, Phuong Hong Nguyen, Spencer Moore, Mandana Arabi

**Affiliations:** ^1^ Department of Health Promotion, Education, and Behavior University of South Carolina Columbia South Carolina USA; ^2^ International Food Policy Research Institute Washington District of Columbia USA; ^3^ Health & Society Group Wageningen University & Research Wageningen Netherlands; ^4^ Nutrition International Ottawa Ontario Canada

**Keywords:** language development, linear growth, motor development, resources for care

## Abstract

**Background:**

Suboptimal child growth and development are significant problems in low‐ and middle‐income countries. Maternal resources for care may help to improve growth and development. This study examined the association of maternal resources for care on child length, motor development and language development of children 12–23.9 months old.

**Methods:**

We used baseline data from the Alive & Thrive household surveys collected in Bangladesh (*n* = 803) and Vietnam (*n* = 635). Resources for care were represented by maternal education, knowledge, height, well‐nourishment, mental well‐being, decision‐making, employment, support in chores and perceived support. The regression analyses were adjusted for household wealth and other covariates on households, children and parents and accounted for geographical clustering.

**Results:**

Maternal height (Bangladesh *β* = 0.150 *p* < 0.001, Vietnam *β* = 0.156 *p* < 0.001), well‐nourishment (Vietnam *β* = 0.882 *p* = 0.007) and mental well‐being (Bangladesh *β* = 0.0649 *p* = 0.008, Vietnam *β* = 0.0742 *p* = 0.039) were associated with child length. Well‐nourishment (Vietnam *β* = 0.670 *p* = 0.042) and support in chores (Bangladesh *β* = 0.0983 *p* = 0.021) were associated with child motor development. Mental well‐being (Vietnam *β* = 0.0735 *p* = 0.013), decision‐making autonomy (Bangladesh *β* = 0.0886 *p* = 0.029) and perceived support (Vietnam *β* = 0.445 *p* = 0.003) were associated with child language development.

**Conclusion:**

Maternal height, well‐nourishment, mental well‐being, decision‐making, support in chores and perceived social support were associated with child outcomes. Interventions that help to improve resources among mothers have potential to foster child growth and development.

**Key messages**

- Poor child growth and suboptimal early childhood development may increase risk of adverse consequences such as poor health and less productivity in later life.
- Resources available to mothers help to shape the environment of the children, consequently impacting child growth and development.
- Maternal height, well‐nourishment and mental well‐being were associated with child length. Maternal well‐nourishment, mental well‐being, decision‐making autonomy, support in chores and perceived support were associated with child development.
- Interventions that aim to improve maternal care resources have potential to positively influence child growth and development.

## 1 INTRODUCTION

Poor child growth and suboptimal early child development remain critical problems in low‐ and middle‐income countries (Black et al., 2013; Black et al., 2017; McCoy et al., 2016). Poor nutritional status decreases immunity power and increases vulnerability to infectious diseases among children (Rice et al., 2000). Additionally, poor nutritional status can restrict the achievement of optimum child development, especially during an early period of life when there is a rapid growth of the brain. Poor nutritional status may also be negatively related to child development by limiting the ability and opportunities of children to be involved in exploration of environment and learning (Prado & Dewey, 2014). Poor development can negatively affect later adult life by influencing educational attainment and productivity (Black et al., 2017; Britto et al., 2017).

Child growth and development may be influenced by multiple conditions in the proximal and distal environment (Frongillo et al., 2017; Maggi et al., 2010). The first and most important context for the provision of care to young children is the home setting, which is often fostered by mothers; therefore, resources available to mothers may be critical for young children (Coller & Kuo, 2015; Engle et al., 1999; Frongillo et al., 2017). The unavailability and inadequacy of resources could constrain mother's capacity to fulfil needs of children and promote their well‐being (Engle, 1999; Engle et al., 1999). The role of mothers is more important in settings that have low accessibility of institutions and programmes that promote nurturing care of children (Borisova et al., 2017; Engle et al., 1999). The extended care model of the United Nations Children's Fund emphasizes that care behaviours and child growth and development are influenced by caregiver's resources such as education and knowledge, physical health, mental well‐being, autonomy, reasonable workload/availability of time and social support. Resources for care improve capabilities and help in meeting children's needs and promoting growth and development (Engle et al., 1997, 1999; Peter & Kumar, 2014). Education and knowledge denote capacity to provide care that is appropriate. Physical and mental health reflects individual‐level resources that help to translate acquired education and knowledge into practices. Autonomy, workload and social support are conditions in family and community that facilitate provision of care (Engle et al., 1997, 1999). These capabilities of and related opportunities for mothers allow them to provide a nurturing environment for their children and can subsequently affect their growth and development (Ickes et al., 2017).

There is a lack of research that has examined the role of multiple measures of resources available to mothers on child length, motor and language development at the same time. Additionally, some resources for care have been studied extensively like education whereas others have received little attention (Engle et al., 1999). This study aimed to examine the association of maternal resources for care with child length, motor development and language development of 12–23.9 months old children in Bangladesh and Vietnam.

## 2 METHODS

### 2.1 Study population

We used the Alive & Thrive baseline data that were collected in Bangladesh and Vietnam in 2010. Alive & Thrive is an initiative that aims to positively impact children's survival, growth and development by improving infant and young child‐feeding practices (Nguyen et al., 2017). Households were selected from 20 subdistricts (*upazilas*) and 40 communes in Bangladesh and Vietnam, respectively. The surveys included mothers and their children less than 5 years of age. Detailed descriptions of the sampling procedure can be found elsewhere (Nguyen et al., 2010; Saha et al., 2011). Ethical approval for the surveys was obtained from the institutional review board of each country and the International Food Policy Research Institute.

In this study, we used data pertaining to mothers and their youngest children 12–23.9 months old (Bangladesh: *n* = 803, Vietnam: *n* = 635). We used this age group because children less than 12 months old may lack significant variation in terms of psychosocial stimulation (Larson et al., 2018), and many children ≥ 24 months would have achieved their motor and language milestones, resulting in little variation (Frongillo et al., 2016).

### 2.2 Measures

#### 2.2.1 Child outcomes

Children's recumbent length was assessed by using locally manufactured collapsible length boards (Nguyen et al., 2010). Length was measured twice by trained personnel, and a third measurement was also taken if the differences between two measurements were significant. The average of the measurements was used for the study (Nguyen et al., 2010).

Language development was assessed using an instrument which was composed of 21 and 20 items related to milestone or skill achievement in Bangladesh and Vietnam, respectively. Motor development was measured using 29 items in both countries (Frongillo et al., 2016). Information on language development was based on reporting from mothers. One point was assigned to each response if a mother indicated that her child had achieved the milestone, and the scores were added to develop an overall language development score (range: Bangladesh 0–21, Vietnam 0–20). Motor development was assessed through mothers' reporting and observation for some items where children were required to demonstrate motor activities. One point was assigned to each affirmative response or observation, and then a score measuring motor development was created by adding the affirmative responses (range: 0–29).

#### 2.2.2 Resources for care

Resources for care were represented by maternal education, knowledge, height, well‐nourishment, mental well‐being, decision‐making autonomy, employment, support in chores and perceived instrumental support. Information on all variables for resources for care was reported by mothers, except the information on physical health which was based on anthropometric measurements.

Maternal education levels differed across our study settings; therefore, we used different cut‐offs based on specific context (Nguyen et al., 2014). In Bangladesh, we categorized education as no schooling and 1–5, 6–9 and 10–12 years of schooling. In Vietnam, we categorized education as 1–5, 6–9 and 10–12 years of schooling and college or higher. Maternal knowledge was specific to the knowledge on breastfeeding, complementary feeding, iron deficiency symptoms, vitamin A sources, iodine fortification, food diversity and hand washing. One point was assigned for each correct response, and the sum was used as a measure of maternal knowledge (total possible scores: 22 for Bangladesh and 23 for Vietnam).

Maternal height and nourishment were used to assess physical health. Nourishment was categorized into well‐nourished (body mass index ≥18.5 kg/m^2^) and underweight (body mass index <18.5 kg/m^2^) (WHO, 2016). Mental well‐being was assessed by using the Self‐Reporting Questionnaire‐20 which is a valid and reliable tool to screen mental disorders in low‐ and middle‐income countries (WHO, 1994). Mental well‐being variable was developed by assigning one point for each item in which absence of symptoms was reported and adding the scores (total possible score of 20).

Decision‐making autonomy was assessed by assigning one point for each item when mothers indicated that they were involved in the decision‐making process alone or along with their spouse or someone else (total possible score of 11). The household decision‐making items were related to major purchases; cooking; visiting family, friends, or relatives; healthcare visit; family planning and care of children including child‐feeding. We did not use financial autonomy as a measure of autonomy because it was closely related to employment. We used employment status (employed vs. not employed) of mothers as one of the measures of autonomy.

Two measures of social support were used: support in household chores and perceived instrumental support. For support in chores, a point was assigned if mothers reported receiving the support and then a summed score was developed (total possible scores: 8 for Bangladesh and 9 for Vietnam). Perceived social support indicated potential instrumental support when in need. A point was assigned when mothers indicated that they would get support if they need money, accommodation, or food. The sum of the scores was used as a measure of perceived instrumental support (total possible score of 3).

#### 2.2.3 Covariates

Household wealth and total number of children < 5 years in the household were household covariates. Household wealth index was constructed using principal component analysis, followed by extracting the first component scores (Vyas & Kumaranayake, 2006). Information used for constructing the household wealth variable was related to house and land ownership, quality of house, access to services (e.g., electricity and cooking fuel) and household assets. Child's age, child's age‐squared, child's age‐cubed, child's gender and father's occupation were used as covariates. Preliminary analysis showed that age to the third power accounted for age trends in the child outcomes.

### 2.3 Statistical analysis

The descriptive statistics were presented as mean and standard deviation (SD) or percentage. Regression models were run to examine the association of maternal resources for care with child length, motor development and language development. Child length and development measures were used in original units adjusted for child age trends and gender to aid comparing the findings across the three outcome measures. Separate models were run for each outcome variable. The models included all measures of resources for care, adjusting for the household, child and parental covariates and accounting for geographical clustering as a random effect. All analyses were conducted using Stata version 14 software.

## 3 RESULTS

### 3.1 Sample characteristics

Mothers in Vietnam had higher education levels than those in Bangladesh (Table 1). Mean maternal knowledge scores were 9.71 ± 2.25 in Bangladesh (maximum possible score = 22) and 9.09 ± 3.01 in Vietnam (maximum possible score = 23). Maternal height, well‐nourishment, mental well‐being, decision‐making autonomy and employment were higher in Vietnam than in Bangladesh. Support in chores had means of 4.60 ± 3.02 in Bangladesh (maximum possible score = 8) and 4.62 ± 3.09 in Vietnam (maximum possible score = 9). Perceived instrumental support was slightly higher in Bangladesh (mean = 2.73 ± 0.760) than in Vietnam (mean = 2.48 ± 0.849) (maximum possible score = 3 for both countries).

**TABLE 1:** Sample characteristics of mothers and children 12–23.9 months old in Bangladesh and Vietnam

	Bangladesh (*n* = 803)	
	Percent or mean ± SD	Vietnam (*n* = 635)
***Maternal resources for care***		
Education, %		
No schooling	27.2	‐
1–5 years schooling	30.1	12.5
6–9 years schooling	34.7	53.5
10–12 years schooling	8.00	21.1
College or higher	‐	12.9
Knowledge (range: B = 0–22, V = 0–23)	9.71 ± 2.25	9.09 ± 3.01
Height, cm	151 ± 5.37	153 ± 5.30
Well‐nourished, %	63.4	69.6
Mental well‐being (range: 0–20)	12.9 ± 5.16	15.0 ± 4.27
Decision‐making (range:0–11)	8.28 ± 2.68	9.02 ± 2.00
Employed, %	6.23	88.8
Support in chores (range: B = 0–8, V = 0–9)	4.60 ± 3.02	4.62 ± 3.09
Perceived support (range: 0–3)	2.73 ± 0.760	2.48 ± 0.849
***Child characteristics***		
Age, months	18.0 ± 3.24	18.0 ± 3.43
Female, %	49.9	45.7
Length, cm	75.7 ± 4.31	78.4 ± 4.76
Motor development (range:0–29)	19.6 ± 3.94	22.1 ± 4.72
Language development (range: B = 0–21, V = 0–20)	9.78 ± 3.38	10.9 ± 3.83
***Household characteristics***		
Household wealth	0.00571 ± 2.71	0.0967 ± 2.44
Number of <5 years children, %		
1 child	79.3	84.9
≥2 children	20.7	15.1
***Father's characteristic***		
Occupation, %		
Unemployed/no husband	2.62	1.40
Farmer	20.1	44.9
Manual or wage‐based work	35.2	13.2
Service or salaried work	14.6	12.3
Business, trade or self‐employed	23.3	28.2
Others	4.18	‐

*Note*: B, Bangladesh; SD, standard deviation; V, Vietnam.

Mean age of children was about 18 months in both countries. About half of the children were females in Bangladesh, whereas 45.7% were females in Vietnam. Child length had means of 75.7 ± 4.31 and 78.4 ± 4.76 cm in Bangladesh and Vietnam, respectively. Motor development had means of 19.6 ± 3.94 and 22.1 ± 4.72 in Bangladesh and Vietnam, respectively (maximum possible score = 29 for both countries). Language development had means of 9.78 ± 3.38 (maximum possible score = 21) in Bangladesh and 10.9 ± 3.83 (maximum possible score = 20) in Vietnam.

More than half of the households had only one child <5 years old in both countries. Most of the fathers were engaged in manual or wage‐based work in Bangladesh, and most of the fathers were farmers in Vietnam.

### 3.2 Association of resources for care with child outcomes

Maternal height was positively associated with child length in both countries (Bangladesh: *β* = 0.150, *p* = <0.001; Vietnam: *β* = 0.156, *p* = <0.001) (Table 2). Children of well‐nourished mothers were taller in Vietnam (*β* = 0.882, *p* = 0.007). Maternal mental well‐being was positively associated with child length in Bangladesh (*β* = 0.0649, *p* = 0.008) and Vietnam (*β* = 0.0742, *p* = 0.0390).

**TABLE 2:** Association of maternal resources for care with length of children 12–23.9 months old in Bangladesh and Vietnam

Maternal resources for care	Child length (cm)					
	Bangladesh			Vietnam		
	*β*	95% CI	*p*‐value	*β*	95% CI	*p*‐value
Education						
1–5 years schooling	Reference	Reference	Reference	Reference	Reference	Reference
No schooling	−0.363	−1.02, 0.297	0.281	‐	‐	‐
6–9 years schooling	0.408	−0.222, 1.04	0.204	−0.851	−1.80, 0.0983	0.079
10–12 years schooling	−0.790	−1.85, 0.274	0.146	−0.386	−1.54, 0.771	0.513
College or higher	‐	‐	‐	−0.565	−2.01, 0.882	0.444
Knowledge	−0.0626	−0.176, 0.0512	0.281	0.0332	−0.0730, 0.139	0.540
Height	0.150	0.105, 0.195	<0.001	0.156	0.100, 0.211	<0.001
Well‐nourished	0.176	−0.339, 0.691	0.503	0.882	0.244, 1.52	0.007
Mental well‐being	0.0649	0.0166, 0.113	0.008	0.0742	0.00377, 0.145	0.039
Decision‐making	−0.00320	−0.0980, 0.0916	0.947	0.00458	−0.150, 0.159	0.954
Employed	0.563	−0.454, 1.58	0.278	0.326	−0.636, 1.29	0.507
Support in chores	−0.0301	−0.117, 0.0569	0.498	0.00133	−0.0969, 0.0996	0.979
Perceived support	0.0883	−0.240, 0.416	0.598	−0.0675	−0.424, 0.289	0.710

*Note*: Coefficients reported in the table are unstandardized. Model adjusted for household wealth, total number of <5 years old in the household, child's age, child's age‐squared, child's age‐cubed, child's gender, father's occupation, and geographical clustering.

Children of well‐nourished mothers (*β* = 0.670, *p* = 0.042) had higher motor development in Vietnam (Table 3). Receiving support in chores (*β* = 0.0983, *p* = 0.021) was positively associated with child motor development in Bangladesh.

**TABLE 3:** Association of maternal resources for care with motor development of children 12–23.9 months old in Bangladesh and Vietnam

Maternal resources for care	Child motor development (milestones achieved)					
	Bangladesh			Vietnam		
	*β*	95% CI	*p*‐value	*β*	95% CI	*p*‐value
Education						
1–5 years schooling	Reference	Reference	Reference	Reference	Reference	Reference
No schooling	−0.0171	−0.650, 0.616	0.958	‐	‐	‐
6–9 years schooling	0.338	−0.264, 0.941	0.271	−0.257	−1.22, 0.703	0.600
10–12 years schooling	0.498	−0.524, 1.52	0.340	−0.0810	−1.25, 1.09	0.892
College or higher	‐	‐	‐	0.602	−0.862, 2.07	0.420
Knowledge	−0.00905	−0.119, 0.101	0.872	0.0582	−0.0492, 0.166	0.288
Height	0.0202	−0.0228, 0.0633	0.357	0.0280	−0.0282, 0.0842	0.328
Well‐nourished	−0.0879	−0.582, 0.406	0.727	0.670	0.0240,1.32	0.042
Mental well‐being	−0.00432	−0.0508, 0.0422	0.855	0.0326	−0.0386, 0.104	0.369
Decision‐making	0.0194	−0.0718, 0.111	0.677	−0.0858	−0.242, 0.0702	0.281
Employed	0.113	−0.850, 1.08	0.818	0.629	−0.344, 1.60	0.205
Support in chores	0.0983	0.0146, 0.182	0.021	0.0334	−0.0660, 0.133	0.510
Perceived support	−0.125	−0.442, 0.192	0.441	0.121	−0.239, 0.481	0.510

*Note*: Coefficients reported in the table are unstandardized. Model adjusted for household wealth, total number of <5 years old in the household, child's age, child's age‐squared, child's age‐cubed, child's gender, father's occupation, and geographical clustering.

Maternal mental well‐being was positively associated with language development in Vietnam (*β* = 0.0735, *p* = 0.013) (Table 4). Children of mothers with higher decision‐making autonomy (*β* = 0.0886, *p* = 0.029) and perceived support (*β* = 0.445, *p* = 0.003) had higher language development in Bangladesh and Vietnam, respectively.

**TABLE 4:** Association of maternal resources for care with language development of 12–23.9 months children in Bangladesh and Vietnam

Maternal resources for care	Child language development (milestones achieved)					
	Bangladesh			Vietnam		
	*β*	95% CI	*p*‐value	*β*	95% CI	*p*‐value
Education						
1–5 years schooling	Reference	Reference	Reference	Reference	Reference	Reference
No schooling	−0.295	−0.849, 0.258	0.296	‐	‐	‐
6–9 years schooling	0.186	−0.340, 0.713	0.488	0.003	−0.782, 0.788	0.994
10–12 years schooling	0.0336	−0.860, 0.927	0.941	0.570	−0.387, 1.53	0.243
College or higher	‐	‐	‐	0.493	−0.702, 1.69	0.419
Knowledge	0.0747	−0.0212, 0.171	0.127	0.081	−0.00673, 0.169	0.070
Height	0.00474	−0.0329, 0.0424	0.805	−0.016	−0.0614, 0.0304	0.507
Well‐nourished	0.0353	−0.397, 0.467	0.873	0.153	−0.374, 0.680	0.570
Mental well‐being	0.00180	−0.0388, 0.0425	0.931	0.0735	0.0153, 0.132	0.013
Decision‐making	0.0886	0.00890, 0.168	0.029	−0.025	−0.152, 0.103	0.701
Employed	−0.457	−1.30, 0.385	0.288	0.403	−0.392, 1.20	0.320
Support in chores	0.0628	−0.0104, 0.136	0.093	−0.062	−0.143, 0.0193	0.135
Perceived support	−0.147	−0.425, 0.130	0.298	0.445	0.150, 0.739	0.003

*Note*: Coefficients reported in the table are unstandardized. Model adjusted for household wealth, total number of <5 years old in the household, child's age, child's age‐squared, child's age‐cubed, child's gender, father's occupation, and geographical clustering.

## 4 DISCUSSION

This study examined the associations of maternal care resources with three distinct measures of early child growth and development in Bangladesh and Vietnam. Maternal height, well‐nourishment and mental well‐being were associated with child length. Well‐nourishment and support in chores received by mothers were associated with child motor development. Maternal mental well‐being, decision‐making and perceived support were associated with language development.

Previous research also has found that mother's height is associated with child growth. Children of taller mothers in India had decreased risk of stunting, underweight and wasting (Subramanian et al., 2009). Positive association of maternal height with linear growth of offspring can be attributed to genetic and nongenetic influences (Addo et al., 2013). Short stature and low body mass index during pregnancy may lead to adverse pregnancy outcomes such as obstructed labour, small‐for‐gestational age and preterm births which may predispose to poor growth and development (Black et al., 2008; Kozuki et al., 2015). Additionally, maternal short stature indicates that mothers grew in a poor and constrained environment (Addo et al., 2013). Short stature may be associated with poor health and economic and educational achievement, which may in turn negatively influence child outcomes (Addo et al., 2013; Dewey & Begum, 2011; Hernandez‐Diaz et al., 1999). Undernourished mothers are more likely to have co‐morbidities and lack of energy which may negatively impact care received by children and subsequent child growth and development (Kulasekaran, 2012).

Consistent with our study, previous research from the low‐ and middle‐income countries found negative associations of poor maternal mental health on children's nutritional status (Nguyen et al., 2014; Nguyen et al., 2018; Surkan et al., 2011). Poor mental health may compromise capacity to care for children, parenting and care practices like breastfeeding and negatively affect children's growth (Holm‐Larsen et al., 2019; Santos et al., 2011; Surkan et al., 2011). Poor mental health may also negatively affect mother–child interactions and attachments. Children of mothers with poor mental health are also more likely to experience low activity level, unresponsiveness and challenges while interacting with unfamiliar adults (Wachs et al., 2009). These factors may explain the positive association of maternal mental health with child language development in our study.

Maternal involvement in household decision‐making was associated with child language development. Higher autonomy among mothers may increase spending of household resources in favour of children (Thomas, 1990). Additionally, mothers are more likely to allocate resources towards their children if they have control over household resources (Quisumbing & Maluccio, 2000).

Support in chores and perceived support had positive associations with motor and language development, respectively. Social support and social capital may help in the provision of a more nurturing environment, which may positively impact children's well‐being (Engle et al., 1999; Harpham et al., 2006). Consistent with our study, Chang found that maternal social support predicted children's language development (Chang, 2017). Mother's social support predicted child language development through improved psychological well‐being among mothers and improved learning environment at home (Chang, 2017).

Some differences in the associations of resources for care with child outcomes occurred between study settings. For example, maternal well‐nourishment was positively associated with motor development only in Vietnam. Previous studies also have suggested that the role of maternal attributes on child outcomes may differ by study settings (Engle et al., 1999; Prado et al., 2017). Maternal attributes and household conditions are crucial for optimal growth and development, but communities, socio‐political contexts and culture may also have influence (Britto et al., 2017). Furthermore, joint effects of selection and causation may occur (Miech et al., 1999). For example, poor mental well‐being among mothers may negatively influence child development and poor child development may further deteriorate maternal mental health.

Three distinct measures of child growth and development and measures of multiple maternal resources for care were used in two different contexts, Bangladesh and Vietnam. Reliable and valid tools were used to the extent possible. We estimated the marginal association of each resource for care with child outcomes adjusting for the other resources for care, which does not estimate the full association of each resource for care with the outcomes. The study was cross‐sectional, limiting causal inference, has limited generalizability for high‐income countries and used self‐reported data for some measures. We lacked data on overall time spent by mothers on leisure and work and could not examine the association of reasonable workload on child outcomes. Although some motor milestones were observed, some reporting biases for child development may have occurred, especially by mothers who were aware of the timing of milestone achievement.

Only some resources for care were important for child growth and development when accounting for other resources for care and covariates. As expected, maternal height was associated with child's length but not with other two outcomes in both countries. Mental well‐being was important for child's length in both countries and for language development in Vietnam but not in Bangladesh. Maternal well‐nourishment was important for child's length and motor development in Vietnam only. Support in chores and maternal decision‐making were important for motor and language development, respectively, in Bangladesh. Perceived social support was important for language development in Vietnam but not in Bangladesh. Taken together, these findings lead to three conclusions. First, having measures of multiple resources for care is important rather than focusing only on one or two as has often been done (Engle et al., 1999). Second, the resources for care most salient for children's development differ by developmental outcome. Third, context matters, with different resources for care most salient in different contexts. Therefore, integrating interventions to improve resources for and capabilities of mothers can best help to improve children's growth and development. Research is warranted to understand the association of resources for care with other child outcomes not examined in this study, especially socio‐emotional development, and to better understand contextual variation in the role of maternal care resources for child growth and development.

## CONFLICT OF INTEREST

No conflict of interest.

## Data Availability

The data that support the findings of this study are openly available in the Alive & Thrive website at https://www.aliveandthrive.org/en/our-data.
